# Supplementary figures and images for: SUMO-dependent transcriptional repression by Sox2 inhibits the proliferation of neural stem cells
Source: PLoS One. 2024 Mar 20;19(3):e0298818. doi: 10.1371/journal.pone.0298818 (PMC10954124; doi:10.1371/journal.pone.0298818)

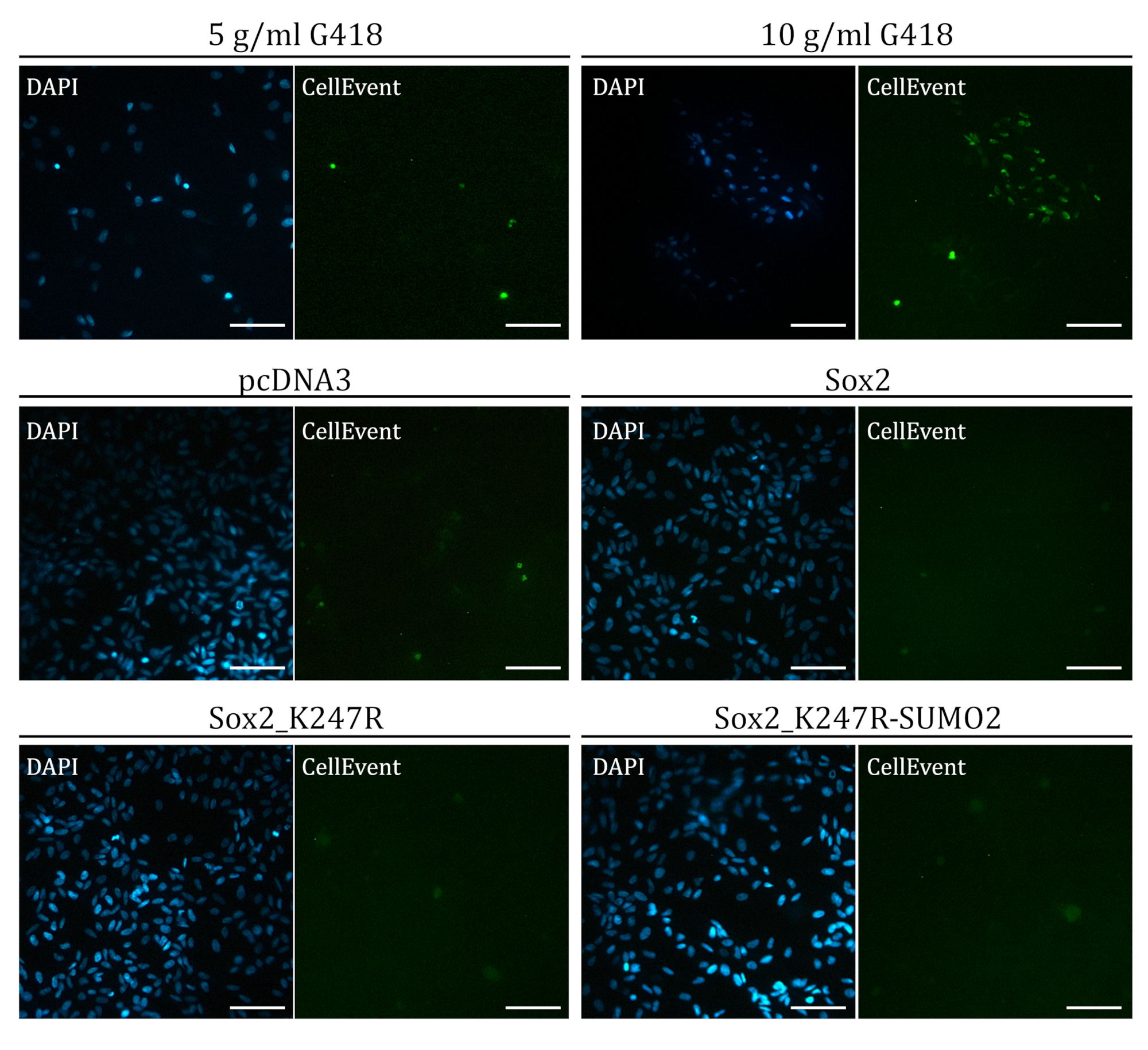

Supplement: S1 Fig — Scale bars: 50 μm. (TIFF) [file pone.0298818.s001.tiff]

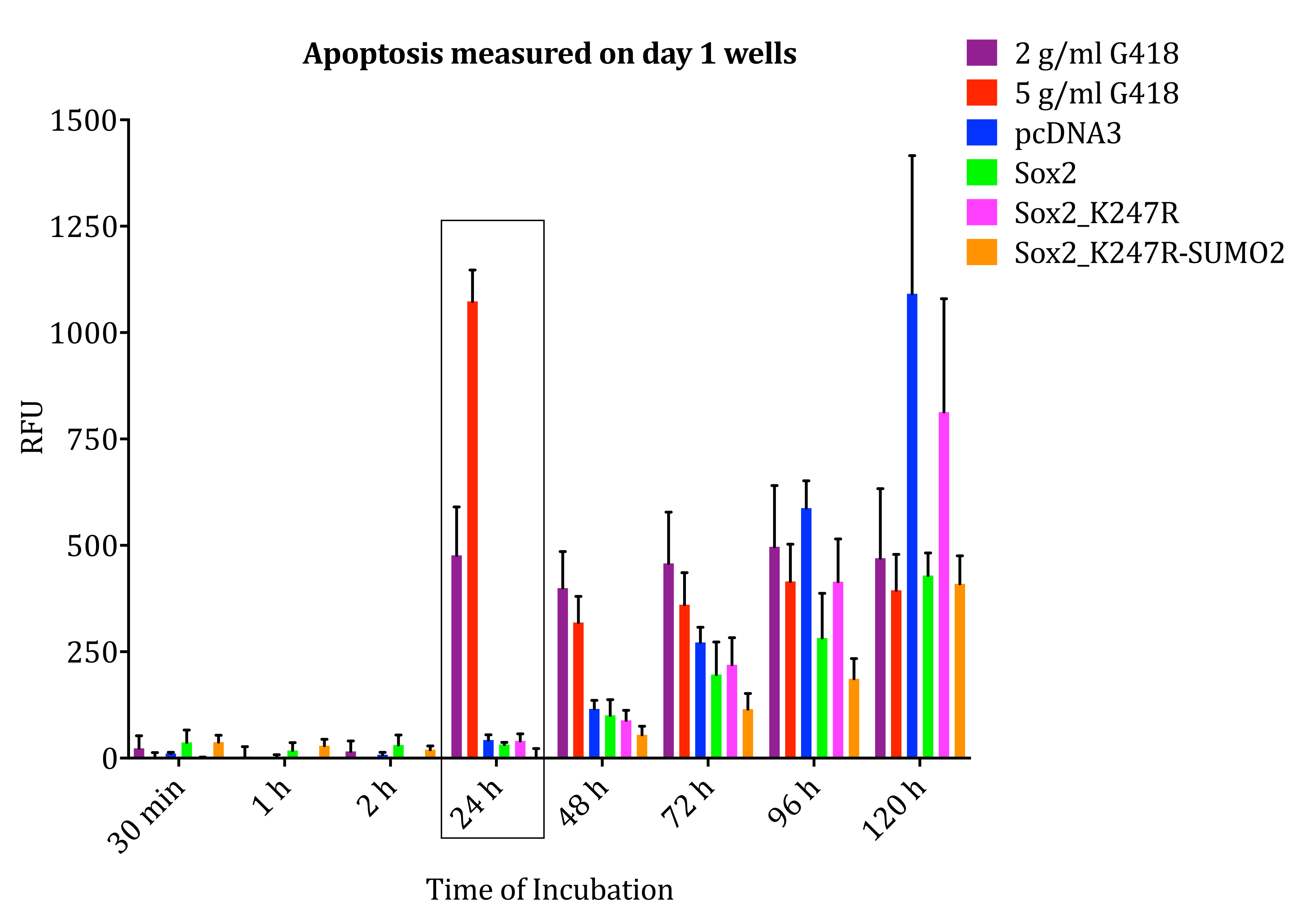

Supplement: S2 Fig — Two biological replicates were performed. Within each biological replicates, two technical replicates were performed. Bars indicate standard deviation of the mean. (TIFF) [file pone.0298818.s002.tiff]

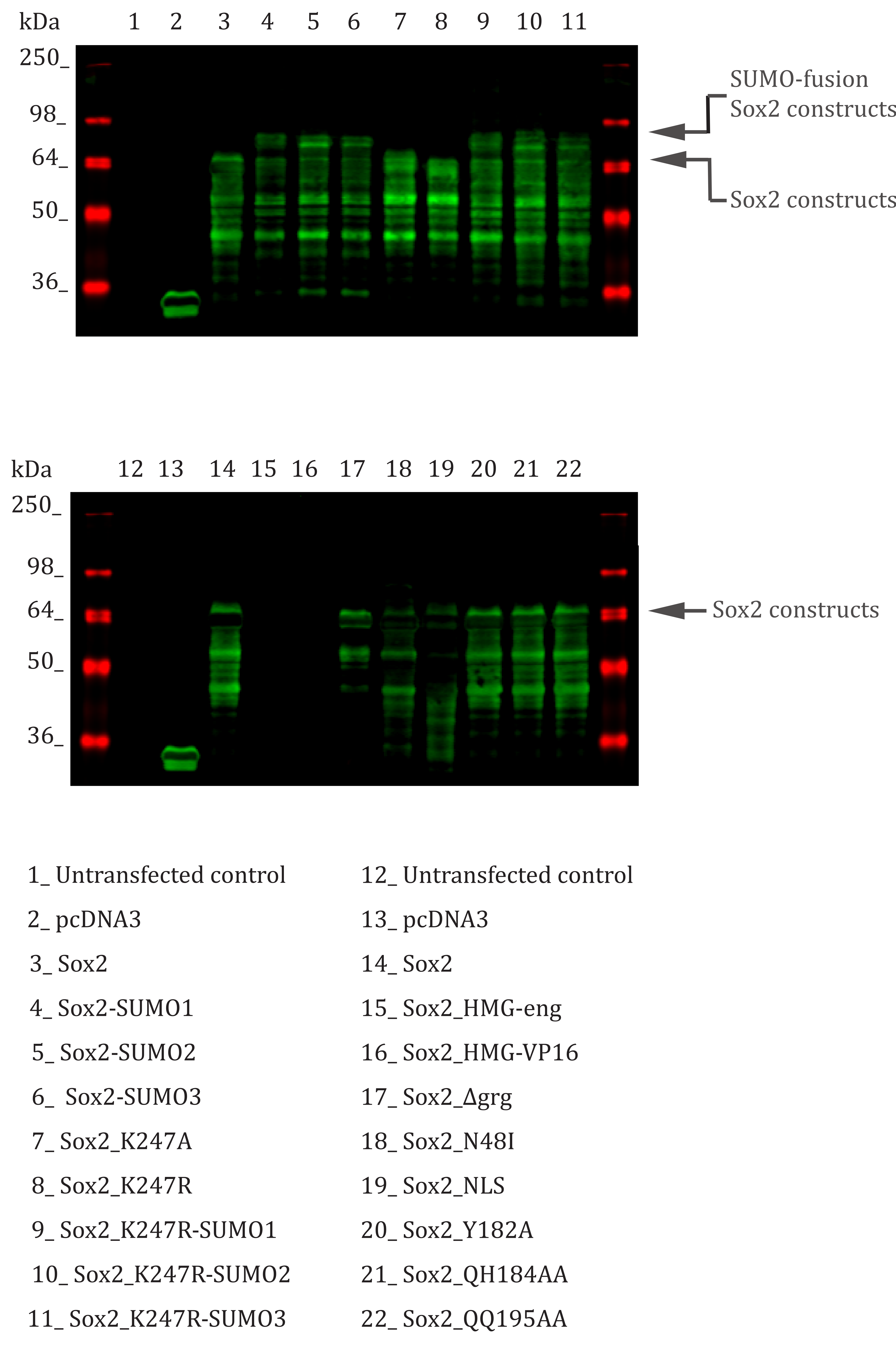

Supplement: S3 Fig — Membrane was probed using anti-Myc primary antibody followed by green Licor secondary antibodies. (TIFF) [file pone.0298818.s003.tiff]

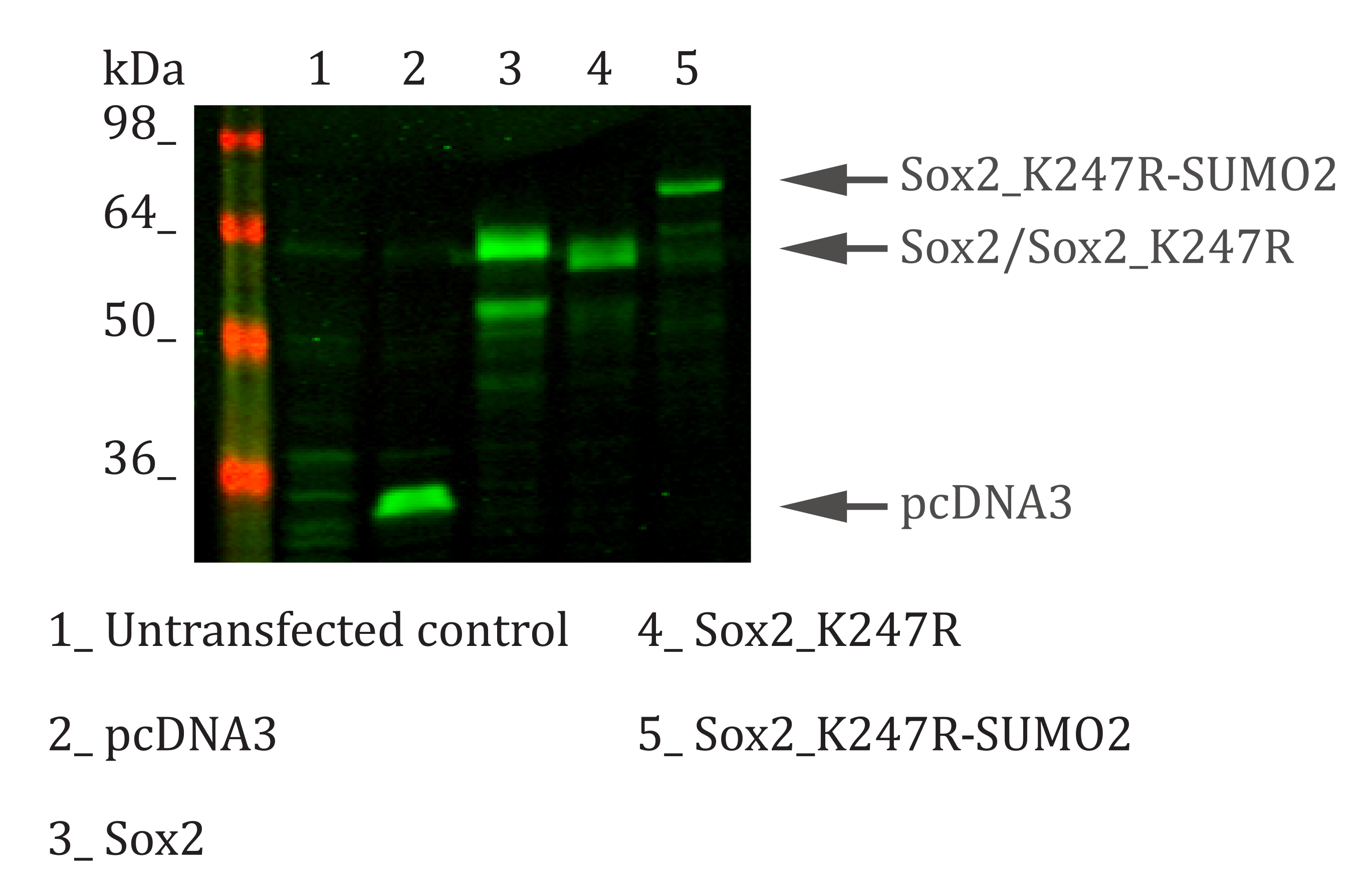

Supplement: S4 Fig — Anti-Myc antibody was used to detect exogenous Sox2 proteins. (TIFF) [file pone.0298818.s004.tiff]

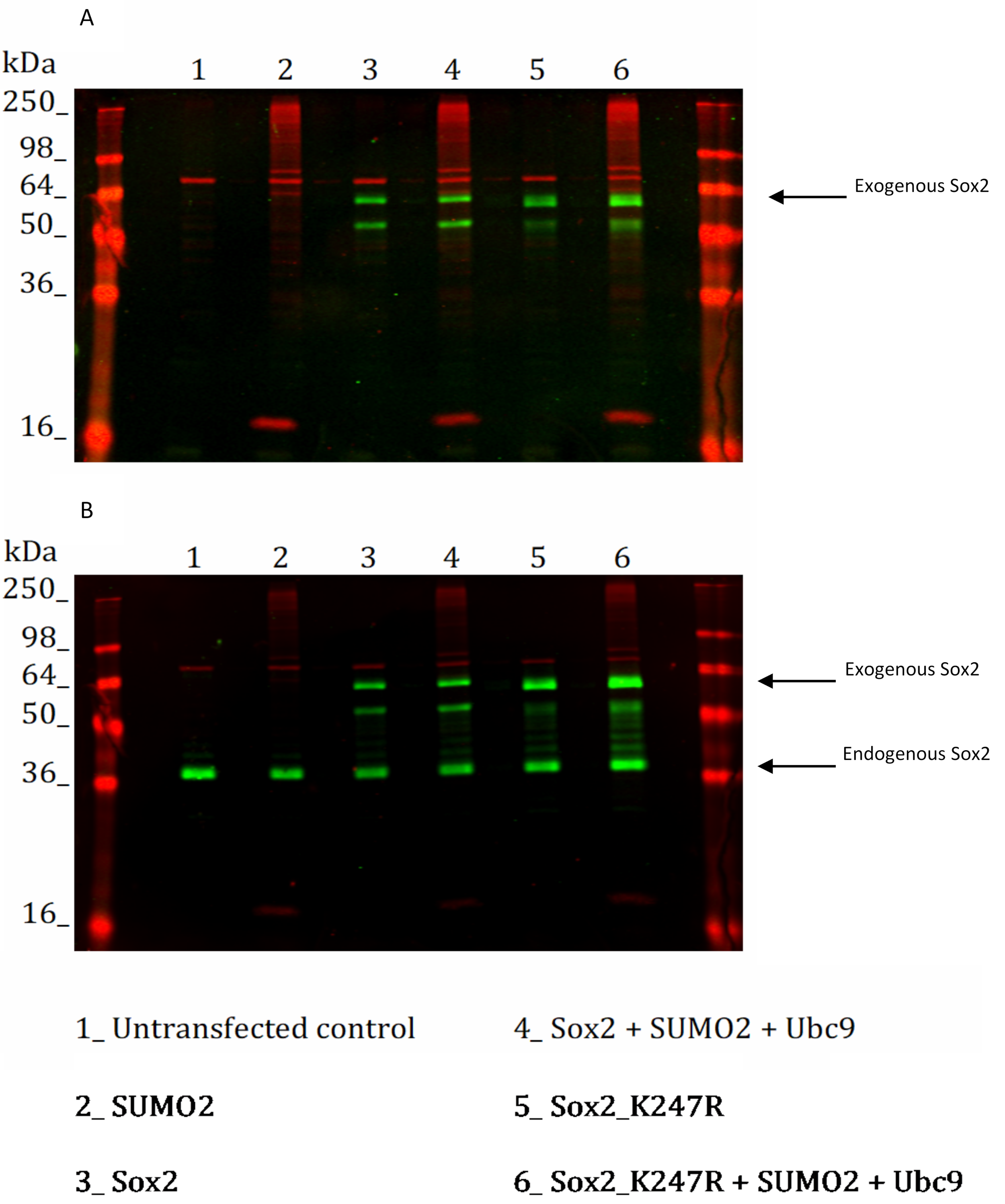

Supplement: S5 Fig — All the Sox2 constructs transfected are Myc-tagged and they are the same constructs used thought the present study. The membrane was firstly probed with mouse anti-Myc and rabbit anti-HA primary antibodies and green LiCor anti-mouse and red LiCor anti-rabbit secondary antibodies (A). The same membrane was then probed again using mouse anti-Sox2 primary antibodies and green Licor anti-mouse secondary antibodies (B). The size difference between exogenous and endogenous Sox2 is due to the exogenous Sox2 constructs containing Myc and His tags. (TIFF) [file pone.0298818.s005.tiff]

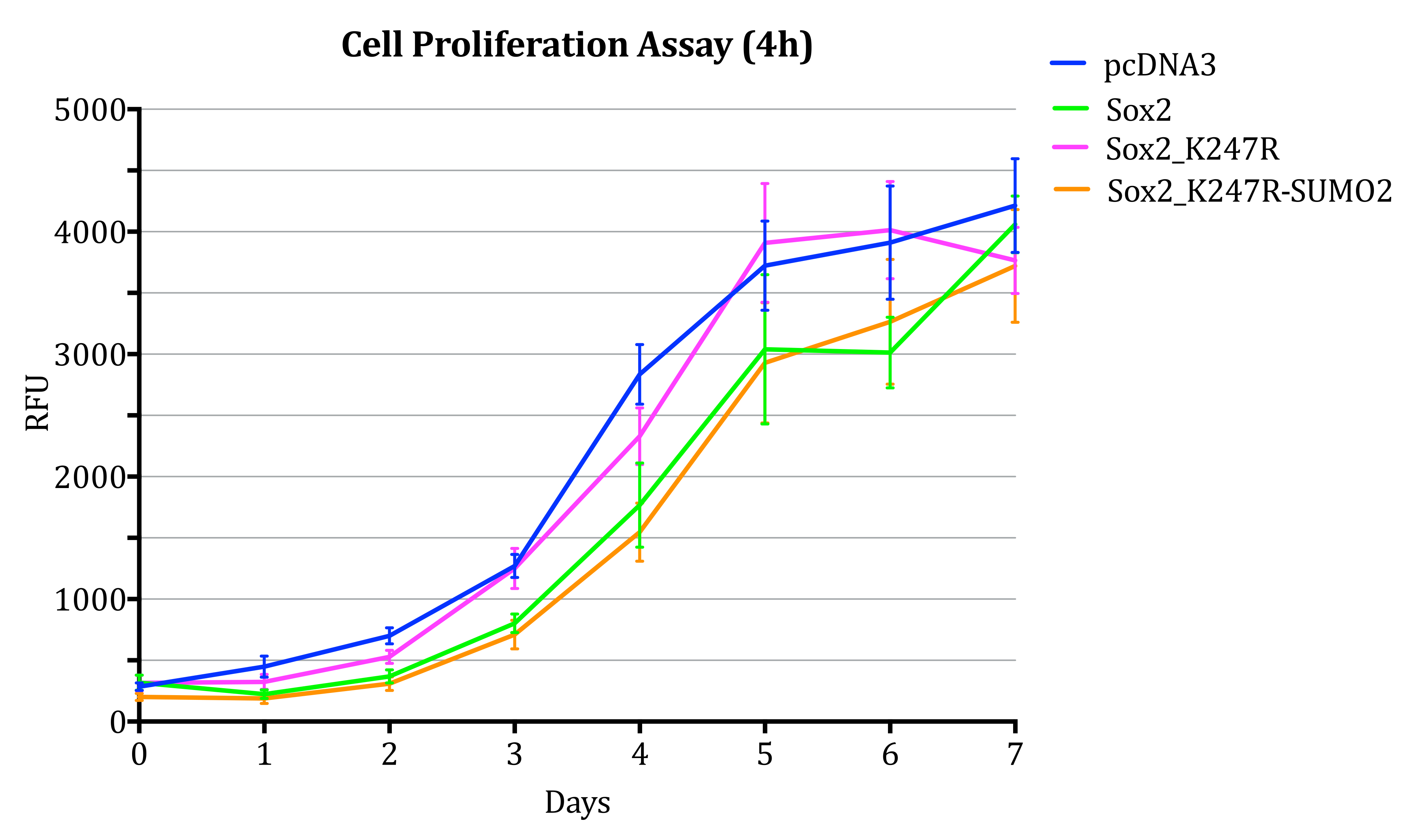

Supplement: S6 Fig — Bars indicate standard error of the mean. (TIFF) [file pone.0298818.s006.tiff]

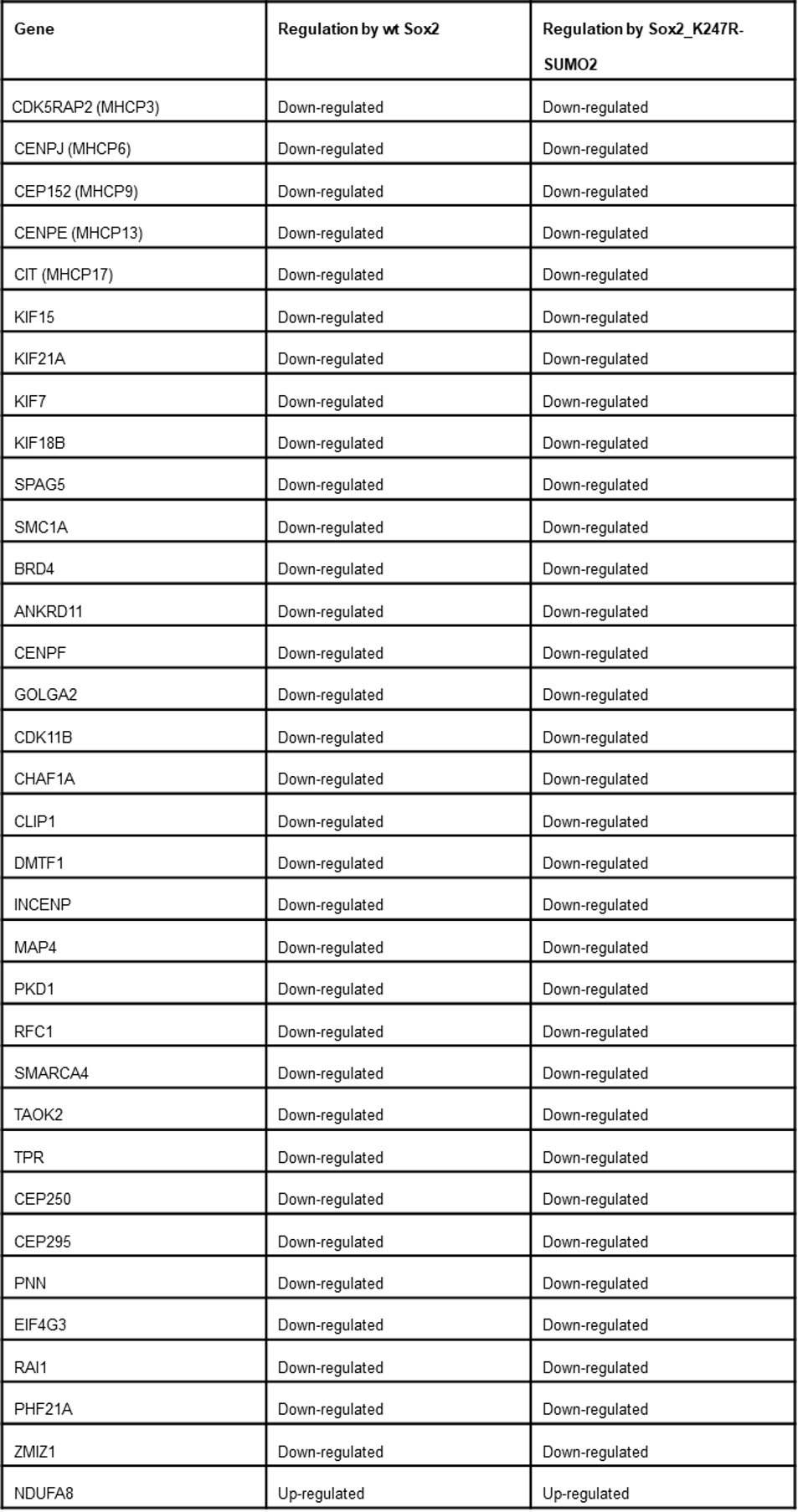

Supplement: S1 Table — (TIF) [file pone.0298818.s007.tif]
